# Prevalence, Trends and Patterns of ADHD Medication Use Among Children, Adolescents and Young Adults in Quebec, Canada: A Repeated Cross-Sectional Population-Based Study From 2000 to 2022: Prévalence, tendances et profils d’utilisation des médicaments pour le TDAH chez les enfants, les adolescents et les jeunes adultes au Québec (Canada) : étude populationnelle transversale répétée, 2000-2022

**DOI:** 10.1177/07067437261471748

**Published:** 2026-08-26

**Authors:** Martin Gignac, Alvine A.K. Fansi, Carlotta Lunghi, Fatoumata Binta Diallo, Louis Rochette, Victoria Massamba, Helen-Maria Vasiliadis, Elham Rahme, Samuele Cortese, Alain Lesage

**Affiliations:** 1 Institut National de Psychiatrie Légale Philippe-Pinel de Montréal, 5599 Université de Montréal, Montreal, Canada; 2 Direction de la Transformation organisationnelle et humaine, Santé Québec, Montréal (QC), Canada; 3 Department of Community Health Sciences, Faculty of Medicine and Health Sciences, University of Sherbrooke, Longueuil, Canada; 4 Department of Life Sciences, Health and Health Professions, 70348 Link Campus University, Rome, Italy; 5 Population Health and Optimal Health Practices Axis, CHU de Québec–Université Laval Research Center, Quebec, Canada; 6 54470 Institut national de santé publique du Québec, Quebec, Canada; 7 Centre de Recherche Charles-Le Moyne, Longueuil, Canada; 8 Department of Medicine, Division of Clinical Epidemiology, 12367 McGill University, Montreal, Canada; 9 Developmental EPI (Evidence synthesis, Prediction, Implementation) Lab, Centre for Innovation in Mental Health, School of Psychology, Faculty of Environmental and Life Sciences, University of Southampton, Southampton, UK; 10 Clinical and Experimental Sciences (CNS and Psychiatry), Faculty of Medicine, 7423 University of Southampton, Southampton, UK; 11 Hampshire and Isle of Wight Healthcare NHS Foundation Trust, Southampton, UK; 12 Hassenfeld Children's Hospital at NYU Langone, New York University Child Study Center, New York City, New York, USA; 13 DiMePRe-J-Department of Precision and Rigenerative Medicine-Jonic Area, University of Bari “Aldo Moro”, Bari, Italy; 14 Département de psychiatrie et d'addictologie, Université de Montréal, Montreal, Canada; 15 Centre de recherche de l'Institut universitaire en santé mentale de Montréal, Montreal, Canada

**Keywords:** child psychiatry, ADHD, medications, pharmacotherapy, drug utilization research, pharmacoepidemiology

## Abstract

**Objective:**

The prevalence of attention-deficit/hyperactivity disorder (ADHD) has risen in recent decades. The objective of this study was to identify trends in prevalence rates of ADHD medication use as well as any patterns of ADHD medication use among individuals aged up to 24 years in Quebec, Canada.

**Method:**

We conducted a repeated cross-sectional study using medico-administrative data between the fiscal years 2000 to 2001 and 2022 to 2023. We estimated crude and age-adjusted prevalence rates along with their 99% confidence intervals according to sex, age (only for crude estimates), material and social deprivation, and ADHD medication type, namely stimulants (amphetamine-based and methylphenidate-based medications) and non stimulants (atomoxetine and guanfacine). We also analyzed prescribing by prescriber type.

**Results:**

Among the 4,771,183 individuals aged ≤24 years, 7.7% claimed ADHD medications. Age-adjusted prevalence of ADHD medication use increased from 2000 to 2001 (1.9%) until 2017 to 2018 (7.9%) and plateaued afterwards. The analysis highlighted not only variations in medication use over time but also by sex, with males more likely to be prescribed these medications than females. Family physicians have become the primary prescribers, overtaking pediatricians in recent years. Stimulant medications were the most common treatment throughout the study period, though we observed diversification in prescribed ADHD medication types across age groups.

**Conclusions:**

This study emphasizes a significant increase in ADHD medication use from 2000 to 2018 and a stabilization in use afterwards. The expansion of diagnostic criteria in 2013 and heightened awareness may have narrowed the disparity between the condition's prevalence and its management and may explain the increase in use observed in earlier years and the stabilization observed over the more recent years. The disparities between males and females, as well as between age groups, highlight the need for continuous monitoring of prescribing trends and support targeted equity evaluations to inform service planning and improve access where needed.

## Plain Language Summary

How has ADHD medication use changed in Québec from 2000 to 2022? changes by age, sex, medication type, prescriber specialty, and neighbourhood deprivation among children, adolescents, and young adults

Attention-deficit/hyperactivity disorder (ADHD) is a common neurodevelopmental condition. Medications can reduce symptoms and are often used alongside non-pharmacological supports. This study describes how ADHD medication use changed over time in Québec among children, adolescents, and young adults. Using health administrative data from the Québec Integrated Chronic Disease Surveillance System, we examined fiscal years 2000-2001 to 2022-2023 among individuals aged 1 to 24 years whose dispensings were observable through coverage under the public drug plan during each year. We estimated the annual prevalence of ADHD medication use and described patterns by sex, age group, neighbourhood material and social deprivation, medication type, and prescriber specialty. ADHD medication use increased steadily from 2000-2001 to 2017-2018 and then stabilized; in 2022-2023, the age-adjusted prevalence was 7.6%. Prevalence was consistently higher among males than females. The highest prevalence shifted over time from children aged 6-11 years to adolescents aged 12-17 years, while use continued to rise among young adults aged 18-24 years. Stimulants remained the most frequently used medications, but prescribing diversified: methylphenidate-based medications became less dominant while amphetamine-based medications became more common. Family physicians became the most frequent prescribers over time, surpassing pediatricians. Prevalence also differed by neighbourhood deprivation, suggesting that socioeconomic context may be linked to patterns of use. These findings provide a long-term picture of ADHD pharmacotherapy in Québec and support continued monitoring to inform service planning and equity-oriented evaluation.

## Graphical abstract

**Graphical abstract:**
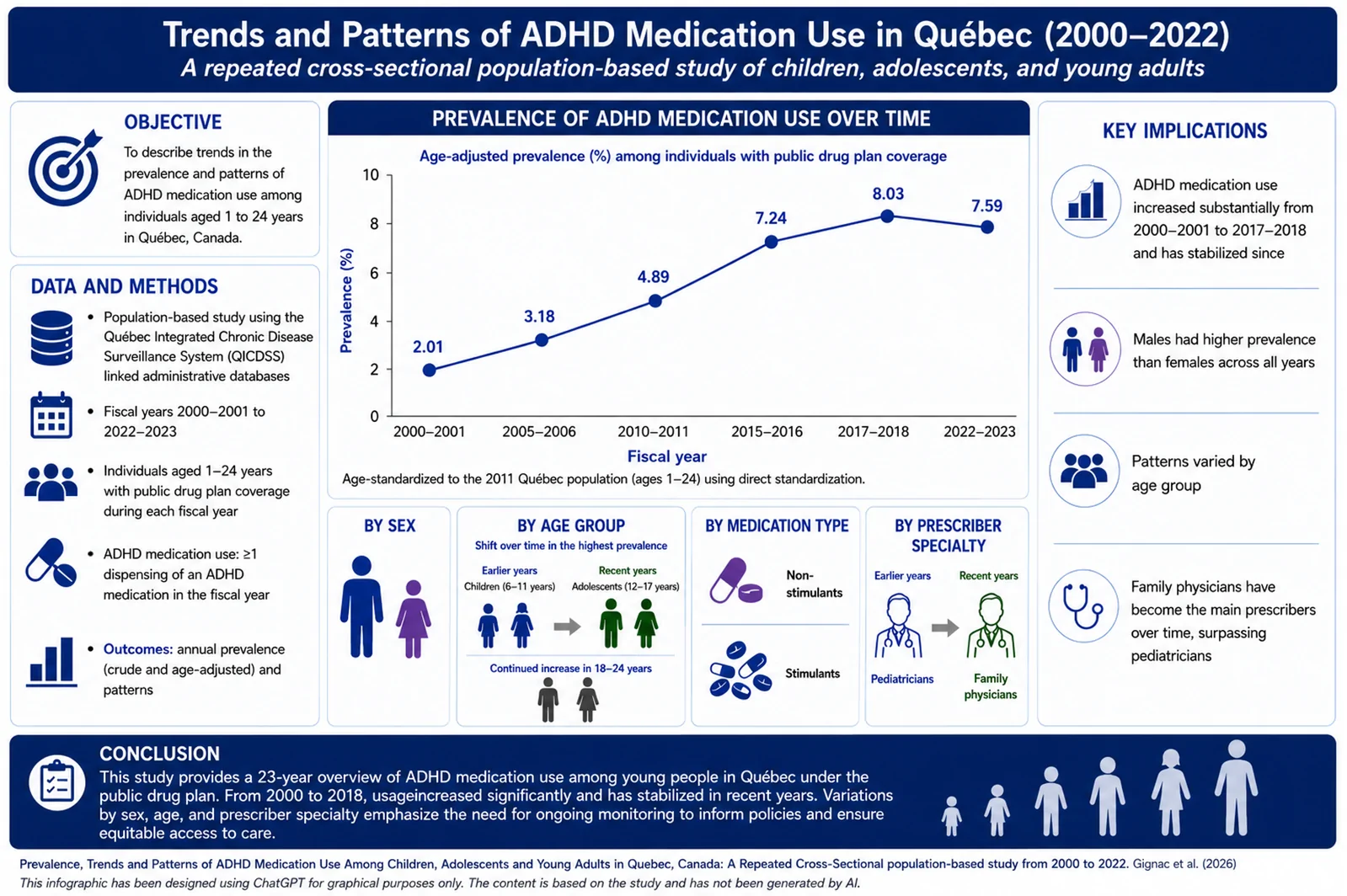
This is a visual representation of the abstract.

## Résumé

**Objectif:**

La prévalence du trouble du déficit de l’attention avec ou sans hyperactivité (TDAH) a augmenté au cours des dernières décennies. L’objectif de cette étude était d’identifier les tendances des taux de prévalence de l’utilisation de médicaments pour le TDAH, ainsi que les profils d’utilisation de ces médicaments, chez les personnes âgées de 24 ans et moins au Québec, au Canada.

**Méthodes:**

Nous avons mené une étude populationnelle transversale répétée utilisant des données médico-administratives des années financières de 2000-2001 à 2022-2023. Nous avons estimé des prévalences brutes et ajustées par l’âge, avec leurs IC à 99%, selon le sexe, l’âge (pour les prévalences brutes), la déprivation matérielle et sociale, et la classe de médicament : stimulants (préparations à base de méthylphénidate ou d’amphétamines) et non-stimulants (atomoxétine ou guanfacine). La répartition des prescriptions a également été analysée en fonction de la spécialité du prescripteur.

**Résultats:**

Parmi les 4 771 183 individus âgés de ≤24 ans, 7,7% ont réclamé au moins un médicament pour le TDAH dans la période. La prévalence ajustée pour l’âge est passée de 1,9% en 2000-2001 à 7,9% en 2017-2018, puis s’est stabilisée. L’analyse a mis en évidence non seulement des variations dans l’utilisation des médicaments au fil du temps, mais aussi selon le sexe, les hommes étant plus susceptibles de se voir prescrire ces médicaments que les femmes. Les médecins de famille sont devenus les principaux prescripteurs, dépassant les pédiatres au fil du temps. Les médicaments stimulants ont été le traitement le plus courant durant toute la période de l’étude, bien que nous ayons observé une diversification des types de médicaments prescrits pour le TDAH selon les groupes d’âge.

**Conclusions:**

Cette étude a mis en évidence une augmentation importante de l’utilisation des médicaments pour le TDAH de 2000 à 2018, suivie d’une stabilisation par la suite. L’élargissement des critères diagnostiques en 2013 et une sensibilisation accrue ont peut-être réduit l’écart entre la prévalence du trouble et sa prise en charge, ce qui pourrait expliquer l’augmentation de l’utilisation observée au cours des premières années ainsi que la stabilisation constatée plus récemment. Les disparités entre les hommes et les femmes, de même qu’entre les différents groupes d’âge, soulignent la nécessité d’une surveillance continue des tendances de prescription et appuient la réalisation d’évaluations ciblées en matière d’équité afin d’orienter la planification des services et d’améliorer l’accès lorsque nécessaire.

## Introduction

Attention-deficit/hyperactivity disorder (ADHD) is a prominent neurodevelopmental disorder frequently diagnosed among children and adolescents, with prevalence estimates ranging from 2% to 8%.^1-6^ A recent meta-analysis estimated the global prevalence of ADHD at 7.6% among 3 to 12-year-old children and 5.6% among those aged 12 to 18,^3^ in line with a critical analysis of the Global Burden of Disease data on ADHD.^7^ While an increase in the trend of ADHD diagnoses has been observed in countries and regions, including Quebec,^4,5,8,9^ it remains debated whether this reflects a true rise in ADHD prevalence or rather changes in diagnostic practices, heightened awareness and evolving clinical criteria. Some evidence suggests that the actual prevalence of ADHD has remained stable over time, with the observed increase in administrative prevalence primarily driven by greater recognition and diagnostic expansion.^10^

ADHD is characterized by core symptoms of inattention, hyperactivity, and impulsivity that can impact various aspects of an individual's life, including familial relationships, school and academic performance, social interactions, professional development, and quality of life.^11-13^ Moreover, ADHD is often associated with psychiatric, neurodevelopmental, and physical comorbidities, as well as a wide array of other negative outcomes, including substance use disorders, school underperformance, unemployment, increased risk of car accident trauma or death, accidental injuries, suicide, and premature death.^2,11,14-24^

Effective management and treatment of ADHD include a multimodal, collaborative approach combining psychosocial interventions with pharmacological treatment.^25-29^ ADHD pharmacotherapy includes stimulants, such as methylphenidate and amphetamines, and nonstimulant medications (atomoxetine and guanfacine).^26,30^ Stimulants are the most commonly prescribed medications and are known for their effectiveness in improving focus and reducing impulsivity and hyperactivity, while non stimulants serve as alternatives, particularly for patients who experience adverse effects from stimulants or have comorbid conditions.^26,30^ The use of ADHD medications has been associated with a reduced risk of unintentional injuries, traumas, mood disorders, conduct disorders, substance-use disorders, attempted or completed suicide, and mortality.^24,26,30-42^ The study in Quebec, which used the same databases as in this study and a within-subjects design, found that episodes of exposure to ADHD medication were associated with a 39% reduction in mortality, 29% reduction in hospitalizations for accidental trauma and 25% reduction in ER visits for accidental traumatic injuries.^33^

In recent decades, there has been a significant rise in ADHD medication prescriptions worldwide, possibly due to changes in diagnostic criteria, greater recognition and response to the disorder, and growing awareness among health-care professionals.^2,43-47^ For instance, a study using population-based prescription databases across multiple countries found that ADHD medication use among children and adolescents increased significantly between 2001 and 2015, with prevalence rates varying from 0.3% in France to 6.7% in the United States.^44^ Another large-scale study analyzing national health records in Canada reported that the proportion of individuals with an ADHD diagnosis receiving pharmacological treatment rose from 39% in 2000 to 62% in 2017,^47^ suggesting improved treatment access, lesser stigma, and potential variations in clinical decision-making.

Importantly, concerns regarding the appropriateness of rising prescription rates must be considered in the context of diagnosed ADHD prevalence. Should the rates of medication prescriptions surpass the estimated prevalence of ADHD, this may indicate a trend of overprescription. Conversely, if prescribing rates are lower, it could suggest that a segment of individuals with ADHD remains untreated, despite the potential therapeutic benefits of pharmacotherapy. In the United States, for instance, state-level estimates from a survey conducted between 2016 and 2019 indicated that 37.8% to 81.4% of children diagnosed with ADHD were receiving ADHD medications, implying that a significant percentage remained untreated.^48^ Less than half of the ADHD diagnosed patients were receiving an ADHD medication in the United Kingdom^49^ or in Germany.^50^ In France, the proportion of children with ADHD-related hospitalizations that were treated with methylphenidate varied between 56.4% and 60.1% in the 2011 to 2019 period, raising concerns about potential undertreatment.^51^ Access to medications may differ not only according to the country, but also to socioeconomic and demographic factors, such as educational level or income.^49,52-54^ Sex is another factor influencing prescribing, with girls being less likely to be diagnosed and treated than boys, a crucial concern that requires attention.^52,55^

Despite extensive international evidence of rising ADHD diagnoses and medication use, province-specific estimates for Quebec have remained fragmented, often limited to pre-2018 horizons, with little attention to young adults, to the type of ADHD pharmacotherapy (stimulant subclasses vs. non stimulants), or to prescriber specialty and socioeconomic gradients. A population-based study reported that the cumulative prevalence of diagnosed ADHD among individuals aged up to 24 years reached 12.6% in 2017 to 2018, making Quebec the province with the highest rate of ADHD diagnoses in Canada.^14^ Understanding whether improved identification and access reflect an increase in treatment or shifts in therapeutic choices is thus essential for service planning and equity monitoring. Moreover, there is a lack of studies that encompass key periods of contextual change (regulatory approvals and updates to clinical guidance, evolving roles for primary care, and system-level reorganization), which could plausibly alter both overall use and its distribution across sex, age, and social and material deprivation strata. Therefore, we designed a population-based, repeated cross-sectional analysis to quantify annual crude and age-adjusted prevalence of ADHD medication use among individuals aged 1 to 24 years; characterize shifts in medication classes and molecules (methylphenidate- vs. amphetamine-based stimulants; atomoxetine; guanfacine); describe prescriber specialty over time; and examine differences by sex, age group, and neighbourhood material/social deprivation from 2000 to 2022.

## Method

### Source of Data and Population

This cross-sectional population-based study draws on linked medico-administrative data sourced from 5 administrative health files belonging to *the Régie de l’assurance maladie du Québec* (RAMQ) and the *Ministère de la Santé et des Services Sociaux du Québec*, which collectively form the Quebec Integrated Chronic Disease Surveillance System (QICDSS). These administrative health files include the public health insurance registry (98% of the 9 million inhabitants of Quebec), the physician claims file, the hospital inpatient and day surgery file (MED-ÉCHO) which identifies the primary and secondary diagnoses related to hospital admission, the vital statistics death database, and the pharmaceutical services database which contains all prescription drug claims for individuals covered by the public prescription drug insurance plan. The study population comprised all children, adolescents, and young adults aged ≤24 years covered by the public health insurance in Quebec, Canada between April 1st, 2000, and March 31st, 2023. Regarding drug insurance in Quebec, individuals may be covered by either employer-based private insurance or the provincial public drug plan (PDP), with coverage depending on their employment status. Approximately 30% of Quebec's younger population is covered by the PDP. It is important to note that prescription drug claims that occurred during private drug insurance plan coverage are not recorded in the QICDSS database. As a consequence, to estimate the prevalence of ADHD medication use, we further excluded all the individuals who were not covered under the PDP at any time during the study period.

### Study Variables and Statistical Analyses

ADHD medications included stimulants (amphetamine-based and methylphenidate-based medications) and non-stimulants (atomoxetine and guanfacine), which were identified from the RAMQ pharmaceutical registry using 5-digit codes specific to the QICDSS internal coding system. These codes are comparable to the 5th level Anatomic Therapeutic Chemical codes. An individual who claimed at least one stimulant or nonstimulant ADHD medication in a specific fiscal year was considered an ADHD medication user in that year. For the purpose of this study, we identified individuals who had full or partial coverage under the PDP for each fiscal year (extending from April 1st of one year to March 31st of the following year) and estimated the prevalence rates among those who were fully covered for the entire year or for a portion of the year, separately and combined.

The crude prevalence of ADHD medication use for a specific fiscal year was determined by dividing the number of ADHD medication users during that year by the PDP-insured population aged 1 to 24 years (i.e., individuals with ≥1 day of PDP eligibility) during that year.

To assess changes over time, age-adjusted annual prevalence rates and 99% confidence intervals were calculated using direct standardization, with the age distribution of the 1- to 24-year-old Quebec population in 2011 as the standard. These prevalence rates were estimated for overall ADHD medication use, as well as by medication class and type. Estimations were also stratified by sex and age groups (1-5 years, 6-11 years, 12-17 years, and 18-24 years), as well as for the area-level social and material deprivation index. This index divided the population into quintiles and was updated using 2016 census data.^56^ The material component is defined by education, employment, and income, while the social component is linked to living arrangements, family structure and marital status. Quintile 1 represents the more advantaged areas and, conversely, quintile 5 represents the most disadvantaged. Analyses were conducted separately for the material and social components.

Different metrics were used to characterize medication choices. First, the annual crude and age-adjusted proportions of stimulant and nonstimulant use were calculated among ADHD medication users only (namely, those with at least one claim for any ADHD medication). Similarly, we calculated the annual proportions of amphetamine, methylphenidate, guanfacine, and atomoxetine use. Second, complementary to the user-based analysis, we also calculated the annual age-standardized distribution of prescription claims. For this analysis, the unit of measurement was the prescription rather than the individual; the numerator consisted of the number of claims for a specific medication class or type, while the denominator was the total number of all ADHD prescriptions claimed during the fiscal year.

For each fiscal year, we further examined the prescriber (classified as family physicians/general practitioners, pediatricians, psychiatrists, or other specialists) associated with their earliest ADHD medication dispensing in that year (the first prescriber of the year) among individuals with at least one ADHD medication dispensing and calculated age-adjusted proportions. This measure identifies the clinician most likely responsible for initiating or maintaining treatment during that year, allowing for year-over-year monitoring of the prescriber. Furthermore, we determined the proportion of ADHD prescriptions attributable to each prescriber specialty, using the total number of ADHD medication prescriptions dispensed to each PDP-insured individual that year as the denominator and the number of ADHD medication prescriptions written by prescribers in the specific specialty as the numerator. We then reported the age-adjusted proportions. Analyses were performed using SAS Enterprise Guide statistical software version 9.4 (SAS Institute).

## Results

We identified 4,771,183 individuals aged ≤24 years who were registered in the QICDSS database at any point between April 1, 2000, and March 31, 2023, with a mean annual cohort size of 2,087,279 individuals. For each fiscal year (i.e., from April 1st to March 31st), PDP eligibility was classified as continuous (365 days) or partial (<365 days). On average, per fiscal year, 555,759 individuals (corresponding to a mean 25.5%) had continuous PDP coverage, and 181,559 (8.3%) had partial coverage. The remainder (1,440,712; 66.1%) were not covered at any time and were excluded from medication dispensing-based analyses. Among those with continuous PDP coverage, a mean of 11,445 (2.1%) had both an ADHD diagnosis (≥1 hospitalization or ≥1 outpatient visit with a diagnostic code for ADHD, namely the international classification of diseases 9th revision [ICD-9] code 314 or ICD-10 codes F900, F901, F908, F909) and ≥1 ADHD medication dispensing, 3,778 (0.7%) had a diagnosis without a medication dispensing, and 19,655 (3.5%) had a medication dispensing without a recorded diagnosis; 520,880 (93.7%) had neither. In the partially covered group, a mean of 2,722 (1.6%) had both; 1,753 (1.0%) had a diagnosis without a medication dispensing; 4,709 (2.8%) had a medication dispensing without a recorded diagnosis; and 172,393 (94.5%) had neither (Supplemental Figure S1). Overall, these distributions correspond to a mean annual total of 38,515 PDP-insured individuals with ≥1 ADHD medication claimed.

**Figure 1.**
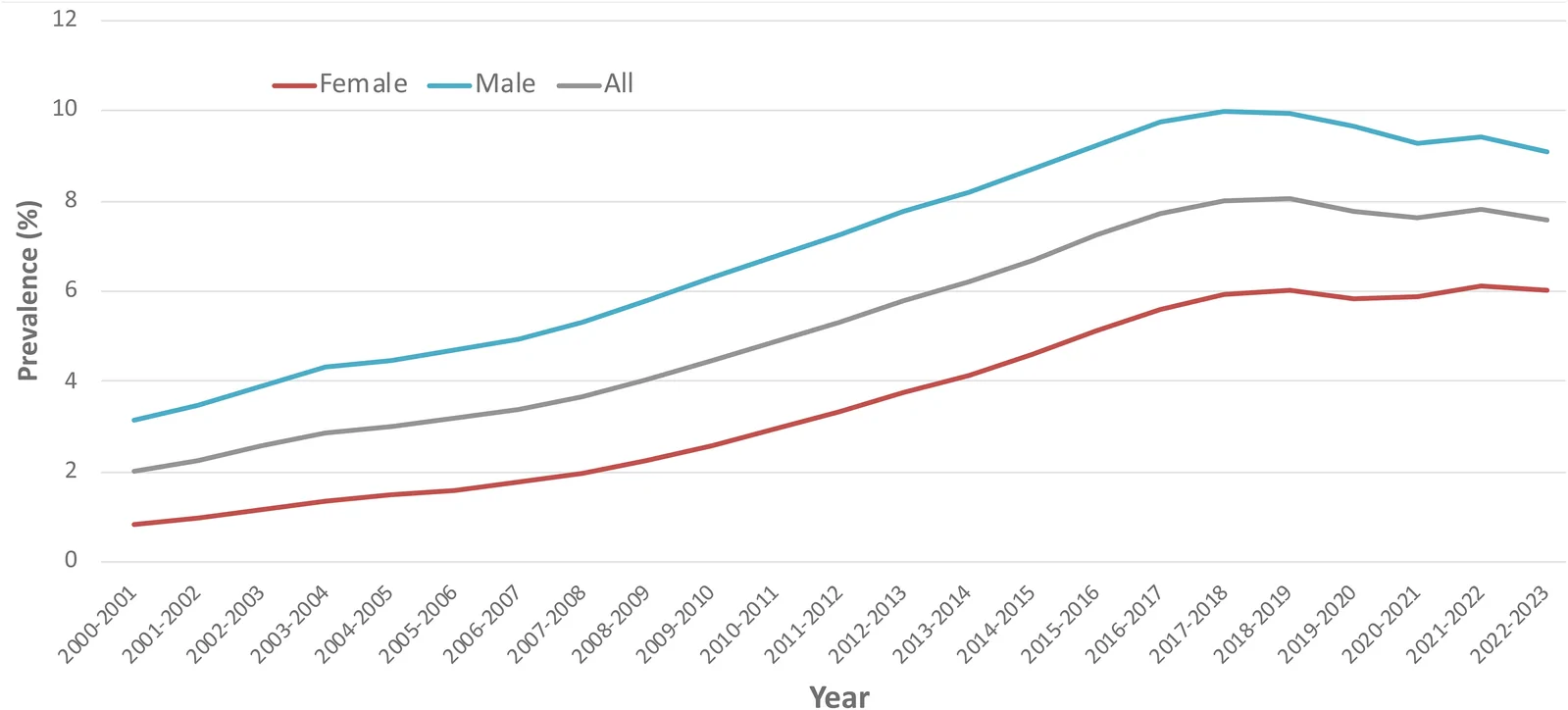
Age-adjusted annual prevalence of ADHD medication use among individuals aged 1 to 24 years fully covered by the public prescription drug insurance plan, by sex, Quebec, from 2000-2001 to 2022-2023.

**Figure 2.**
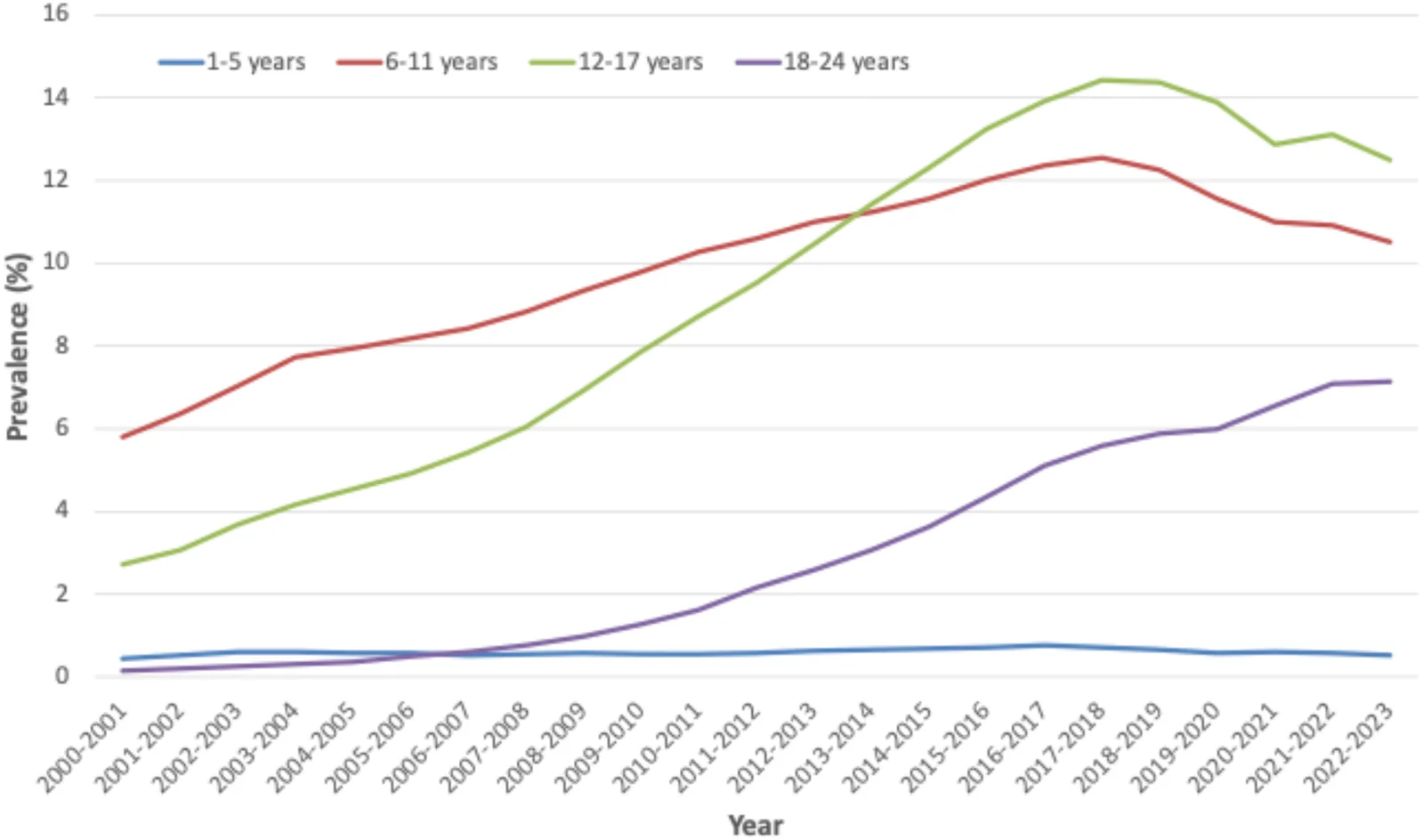
Annual prevalence of ADHD medication use among individuals aged 1 to 24 years, fully covered by the public prescription drug insurance plan, according to age groups, Quebec, from 2000-2001 to 2022-2023.

The trend of age-adjusted annual prevalence of ADHD medication use showed a consistent increase from 2000-2001 to 2017-2018, rising from 1.9% to 7.9% in the overall yearly cohort (Table 1). In the subsequent years, the prevalence rates of medication use remained relatively stable, reaching 7.6% in 2022 to 2023. The prevalence rates of ADHD medication use also varied significantly by sex, with males consistently exhibiting higher prevalence rates than females. However, similar patterns were observed over time for both sexes as depicted in Figure 1. The prevalence of ADHD medication use varied based on age groups, with the 6 to 11 age group having the highest prevalence rates in the 2000s until 2013 to 2014, and the 12 to 17 age group experiencing the highest rates since then (Figure 2). Notably, there were distinct patterns across these age groups, with the 12 to 17 age group experiencing the largest increase through 2017 to 2018, followed by a decline. In contrast, the 18 to 24 age group continued to show a steady increase in prevalence also after 2017 to 2018 (Figure 2).

ADHD = attention-deficit/hyperactivity disorder.

ADHD = attention-deficit/hyperactivity disorder.

**Table 1.** Age-adjusted Annual Prevalence of ADHD Medication use Among Individuals Aged 1 to 24 Years, by Provincial Public Drug Plan (PDP) Coverage, Québec, From 2000-2001 to 2022-2023.^α^

	All*				Total PDP Coverage in the Year (Without Interruptions)				Partial PDP Coverage in the Year (With Interruptions)			
Year	Users	Prevalence	99% CI		Users	Prevalence	99% CI		Users	Prevalence	99% CI	
2000-2001	17,115	1.92	1.89	1.96	13,970	2.01	1.97	2.06	3140	1.62	1.55	1.70
2001-2002	18,975	2.17	2.13	2.21	15,250	2.25	2.20	2.30	3720	1.91	1.83	1.99
2002-2003	21,315	2.49	2.45	2.54	17,020	2.57	2.52	2.62	4295	2.26	2.17	2.35
2003-2004	23,080	2.75	2.70	2.80	18,610	2.86	2.80	2.91	4470	2.40	2.31	2.50
2004-2005	23,740	2.86	2.81	2.91	19,325	3.00	2.95	3.06	4415	2.40	2.31	2.50
2005-2006	24,415	3.00	2.95	3.05	20,245	3.18	3.13	3.24	4170	2.36	2.27	2.46
2006-2007	25,475	3.15	3.10	3.21	21,140	3.38	3.32	3.44	4335	2.41	2.32	2.51
2007-2008	26,840	3.42	3.36	3.47	22,275	3.66	3.60	3.73	4570	2.61	2.51	2.71
2008-2009	29,000	3.79	3.74	3.85	23,640	4.06	3.99	4.13	5360	2.98	2.88	3.09
2009-2010	31,575	4.23	4.17	4.29	25,895	4.47	4.39	4.54	5675	3.47	3.35	3.59
2010-2011	34,130	4.63	4.56	4.69	27,690	4.89	4.81	4.97	6440	3.80	3.68	3.92
2011-2012	36,695	5.06	4.99	5.12	29,800	5.31	5.23	5.39	6900	4.23	4.10	4.37
2012-2013	39,965	5.57	5.49	5.64	32,325	5.78	5.70	5.87	7640	4.81	4.67	4.96
2013-2014	42,855	6.02	5.94	6.10	34,680	6.22	6.13	6.31	8175	5.29	5.13	5.45
2014-2015	46,345	6.50	6.43	6.58	37,345	6.68	6.60	6.78	9000	5.80	5.64	5.97
2015-2016	50,390	7.07	6.98	7.15	40,695	7.24	7.15	7.34	9695	6.34	6.17	6.51
2016-2017	54,625	7.59	7.50	7.67	43,940	7.73	7.63	7.83	10,680	6.97	6.79	7.15
2017-2018	57,235	7.91	7.82	7.99	45,775	8.03	7.93	8.13	11,460	7.38	7.20	7.57
2018-2019	57,590	7.97	7.89	8.06	45,925	8.04	7.94	8.14	11,665	7.66	7.48	7.85
2019-2020	56,080	7.81	7.73	7.90	44,705	7.79	7.69	7.89	11,380	7.82	7.63	8.01
2020-2021	54,520	7.74	7.65	7.83	43,710	7.62	7.52	7.71	10,805	8.20	7.99	8.40
2021-2022	56,770	7.83	7.75	7.92	45,550	7.81	7.72	7.91	11,220	7.88	7.68	8.07
2022-2023	57,105	7.64	7.55	7.72	45,790	7.59	7.50	7.68	11,320	7.74	7.55	7.93

ADHD = attention-deficit/hyperactivity disorder; QICDSS = Quebec Integrated Chronic Disease Surveillance System.

*The “All” column includes those with partial and full coverage to the PDP in the fiscal year.

α All numerators and denominators were randomly rounded to multiples of 5, as is commonly done when using data from the QICDSS.

Supplemental Figure S2 depicts the prevalence of ADHD medication prescribing in accordance with the material and social deprivation index at birth. The findings indicate a higher prevalence of use among individuals living in areas with higher levels of material deprivation, with these disparities becoming more pronounced in recent years. The data did not show substantial differences in prevalence by social deprivation across earlier years. However, from 2017 onward, differences between the most and the least deprived quintiles were observed.

**Figure 3.**
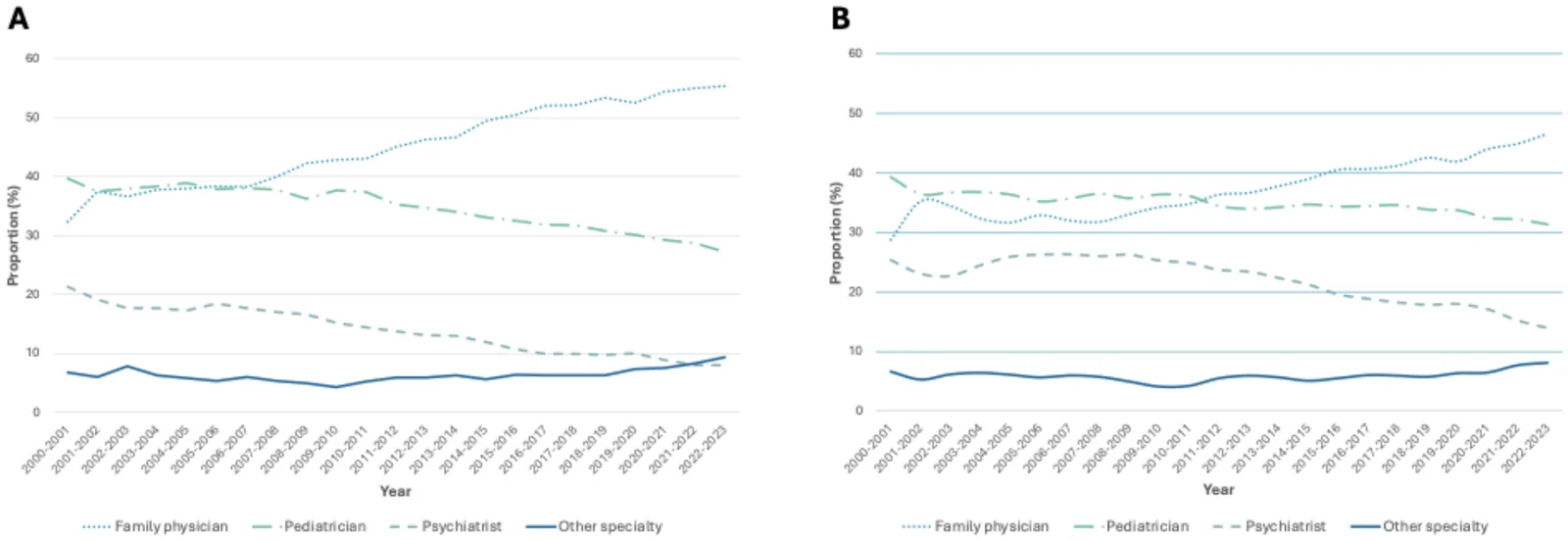
Prescriber specialty indicators for ADHD pharmacotherapy, Quebec, fiscal years 2000-2001 to 2022-2023. (A) Age-standardized distribution of the “first prescriber of the year” among ADHD medication users aged 1 to 24 years (%). Denominator: users with ≥1 dispensing in the fiscal year. (B) Age-standardized proportion of ADHD prescriptions by prescriber specialty (%). Denominator: total ADHD prescriptions in the fiscal year. Estimates pertain to individuals fully covered by the public prescription drug insurance plan, aged 1 to 24 years. *Note:* denominators differ across panels; measures are not directly comparable.

Family physicians and pediatricians were the primary prescribers of ADHD medications, with family physicians being the most frequent prescribers since 2011 to 2012 (Figure 3(B)).

ADHD = attention-deficit/hyperactivity disorder.

**Figure 4.**
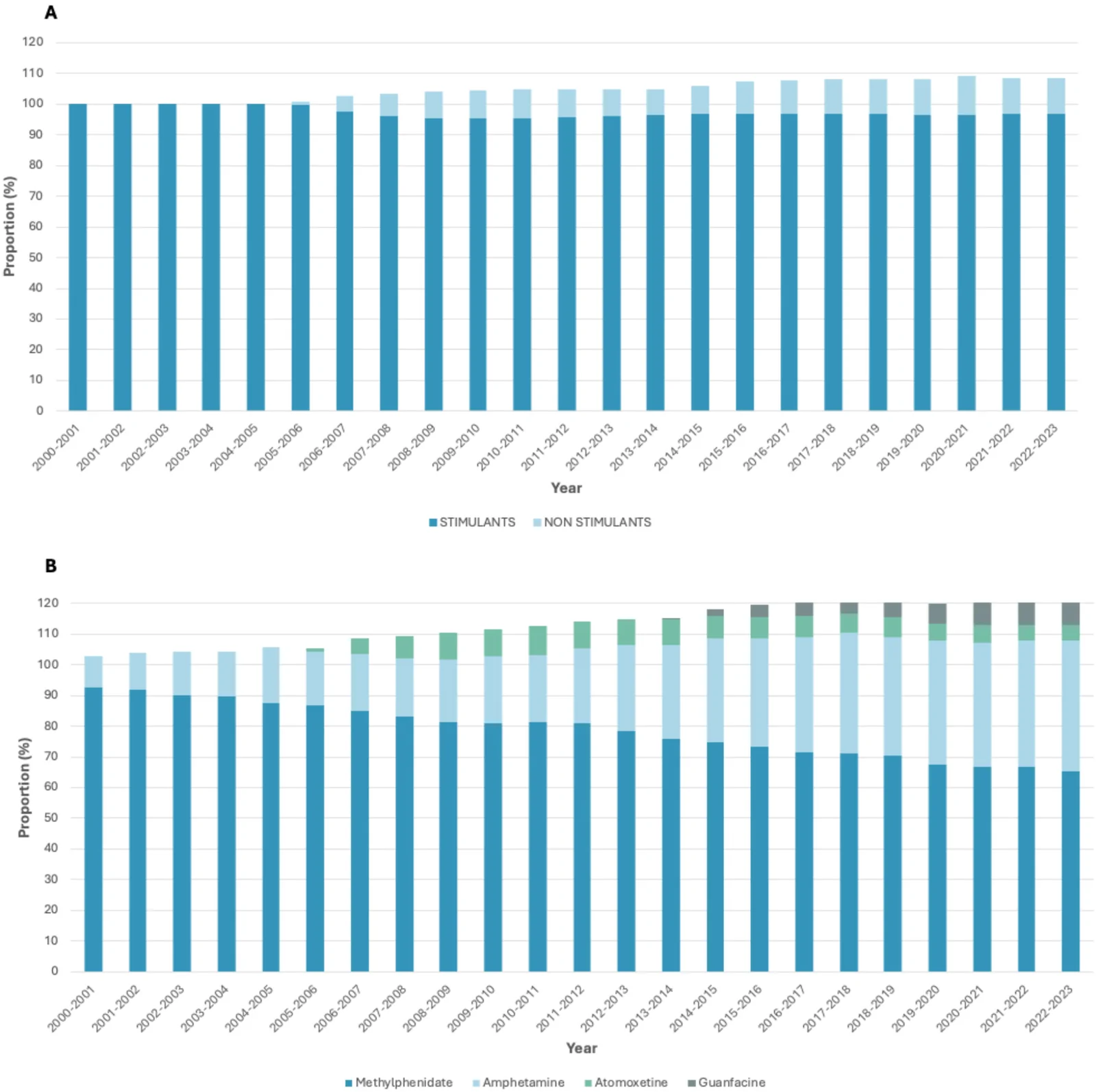
Age-adjusted proportions of ADHD medication use among those with at least one claim in the fiscal year who were fully covered by the public prescription drug insurance plan. (A) Trends by ADHD medication class (stimulants vs. non stimulants) and (B) by ADHD medication type (methylphenidate-based, amphetamine-based, atomoxetine-based, and guanfacine-based medications). Note: the numerator is the number of users of the specific ADHD medication class or type in the year, while the denominator is the total number of users of any ADHD medication in that year. Percentages can exceed 100% because an individual may claim more than one medication class or type.

The most commonly prescribed ADHD medications were stimulants, with non stimulants first prescribed in 2005 to 2006, with a slight increase each year afterward (Figure 4(A) and Supplemental Figure S3(A)). When we stratified the analyses by medication classes and individual molecules, we identified interesting patterns (Figure 4(B); Supplemental Figure S3(B)). Methylphenidate-based medications were the most frequently prescribed during the study period; however, their prevalence among users decreased from 93% to approximately 65%. On the other hand, amphetamine-based medications showed an increase from around 10% to 42%. Among nonstimulant medications, atomoxetine, a selective noradrenaline reuptake inhibitor, has been prescribed since 2005 to 2006, with prevalence rates among users increasing until 2010 to 2011, then slowly decreasing and stabilizing thereafter. In contrast, guanfacine, an α2a agonist medication, has been prescribed since 2014 to 2015, following its approval by Health Canada in 2013, with a slightly increasing prevalence observed during this period. Age-standardized distributions of ADHD prescriptions by ADHD class and medication type are presented in Supplemental Table S1. Different prescribing patterns were also identified by medication class or type, by sex and age groups (Supplementary Figures S4-S6). Particularly, sex gaps are no longer present in the 18 to 24 years groups, especially for stimulants (Supplemental Figure S5), where prevalence rates between males and females have converged. By 2022 to 2023, the prevalence of amphetamine and methylphenidate use among young adult females was comparable to that among males. In contrast, sex differences persisted within the nonstimulant class (Supplemental Figure S6), where alpha-2 adrenergic agonists were prescribed more often to young adult males than to females, whereas atomoxetine use showed a narrower sex distribution.

ADHD = attention-deficit/hyperactivity disorder.

## Interpretation

To our knowledge, this is one of the most extensive repeated cross-sectional population-based studies monitoring the annual prevalence of ADHD medication use over 2 decades among children, adolescents, and young adults aged 1 to 24 years. This study showed an increase in ADHD medication use during the study period, with a plateau reached in 2018 to 2019 (reaching 8.0% among patients with total PDP coverage) and a slight decrease in 2022 to 2023 (7.6%). The prevalence observed in 2022 to 2023 is of a similar order of magnitude to ADHD diagnosis estimates among Quebec youth, although these measures are not directly comparable.

As expected, ADHD medication use was more prevalent in males than in females. This was consistent with clinical samples, with more boys being treated for ADHD compared to girls.^57^ This difference may be due to boys being identified earlier, as they tend to exhibit more hyperactive/impulsive symptoms compared to girls, who typically exhibit more inattentive symptoms without hyperactivity-impulsivity.^58^ Furthermore, it is common for female patients to be diagnosed later on, typically when they exhibit comorbid conditions such as anxiety disorders.^59^ Therefore, it is crucial to provide education and training to practitioners regarding female ADHD. Moreover, evidence regarding ADHD among nonbinary and gender-diverse individuals is limited, and gender identity is not captured in our administrative data. This prevents estimation of ADHD medication use in these groups and may mask disparities in access or treatment. Future work should incorporate measures of gender identity and assess potential differences in diagnosis, prescribing, and continuity of ADHD pharmacotherapy.

The socioeconomic background, as measured by the material deprivation index, showed that highly advantaged families may use fewer ADHD medications (Supplemental Figure S2(A)). In contrast, with the social deprivation index, the gradient reversed: youth living in socially advantaged areas showed a higher prevalence of ADHD medication use than those in socially deprived areas across most years (Supplemental Figure S2(B)). This divergence between material and social dimensions suggests different mechanisms, such as social capital, care-seeking, and service availability, that may shape access to pharmacotherapy and should be considered when interpreting equity patterns. Allied health professionals, including psychologists, occupational therapists, speech-language pathologists, and behaviour therapists, can provide psychosocial interventions (e.g., parent management training, cognitive-behavioural therapy, and school-based interventions) that can complement pharmacotherapy.^60,61^ In Quebec, access to these services through the public system is often limited or episodic, with long wait times; sustained care typically requires private services and out-of-pocket payments. Consequently, families with greater financial resources are more likely to obtain ongoing nonpharmacological care, which may partly explain lower reliance on medication in materially advantaged groups. Expanding publicly funded, multidisciplinary ADHD services could potentially mitigate these socioeconomic differences in treatment options.

In conclusion, the mental health care system in Quebec is characterized by a robust first line of support, as evidenced by the increasing number of primary care physicians who prescribe ADHD medications. Although this approach may differ in other parts of the world, where only specialists are allowed to initiate ADHD medication, Quebec's system provides broader access to these medications. To maintain the effectiveness of this system, it is crucial to provide ongoing medical education to primary care physicians, equipping them with the necessary tools to assess families seeking consultation for suspected ADHD in their children. Furthermore, psychoeducation directed to children and their families is crucial to successful treatment outcomes.

The results of this study must be taken in the context of a number of limitations. The QICDSS is a set of linked administrative health databases that provides useful information on the health status of Quebecers and is largely used for research purposes.^62^ However, diagnostic codes available in physician claims and hospital data do not permit valid classification of DSM ADHD presentation/subtype; therefore, subtype-specific trends could not be assessed. Additionally, the absence of a diagnostic code does not necessarily mean an individual is ADHD-free, as several factors may lead to underreporting. Diagnostic codes are not mandatory in the fee-for-service billing database, and their use has generally declined since 2000 to 2001, especially after a 2016 billing system change. In 2022 to 2023, across the entire database, 65% of billings had a diagnostic code, 6% were coded as “diagnosis missing or unspecified,” and 29% were left blank. Moreover, the pharmacy services file that makes up the QICDSS contains only filled prescriptions dispensed to individuals covered by the PDP, while medications purchased by individuals covered by an employer-based private prescription drug insurance plan (or by their parents if minors) are not recorded in the QICDSS database. Also, we did not assess dosing levels, titration patterns, or concordance with clinical guidance. Dose-level evaluation would require additional dispensing details and clinical context that were not available or not within scope for this surveillance analysis. The PDP covers approximately 30% of the younger Quebec population. Since the population covered by private insurance may differ from that covered by the PDP, we cannot assume that the prevalence rates we found apply to the total population of Quebec. Individuals under a private drug plan are generally more materially advantaged and may have easier access to psychotherapeutic approaches that are not reimbursed by the RAMQ. However, our results are very similar to those of the *Institut national d’excellence en santé et services sociaux*, which used estimates from IMS Health (now IQVIA) from community pharmacy data (covering about 74.3% of the prescriptions in Quebec) (https://www.iqvia.com/-/media/iqvia/pdfs/canada/fact-sheets/iqvia_2023_snapshot_psychostimulants_2019-2022.pdf). In that report based on community pharmacy data, the annual prevalence of ADHD stimulant dispensing in 2021 was 11.2% among boys aged 0 to 18, whilst a separate analysis of PDP for boys aged 1 to 17 gave a very similar figure of 11.5% (data not reported): this suggests that the non-PDP covered are being prescribed similarly and the PDP figures are a good proxy for the whole population, at least for the ADHD youth population.

The current findings confirm the increasing prevalence of ADHD medication use over time. This trend of increasing prescribing is consistent with the trend of ADHD diagnosis in many countries.^43,44,63^ The proportion of people who received an ADHD medication prescription differs by age and sex. These disparities likely reflect factors beyond access alone, including sex- and age-specific differences in diagnostic recognition and symptom profile, longer treatment duration and persistence with increasing age (i.e., cumulative exposure), comorbidity/contraindication considerations in treatment selection, preferences for nonpharmacological management, and risk–benefit perceptions (e.g., adverse effects or diversion concerns in adolescents).^44,57,64^

The information from this study provides a comprehensive picture of the annual patterns of ADHD medication use in Quebec from 2000 to 2022. These monitoring results can contribute to a better understanding of the potential effects of healthcare and medication use for ADHD. These results also inform decision-makers and stakeholders in defining priorities and courses of action, developing appropriate policies, and implementing services that meet the needs of the population. Finally, this study also provides many avenues for future research. Additional analyses are needed, particularly to describe the short- and long-term association between ADHD medication use and health outcomes, as well as medical service use in the ADHD population. The findings from this research can provide a comprehensive overview of ADHD pharmacotherapy practices over 2 decades, detailing therapeutic classes and patterns by sex, age, and material deprivation, and highlighting potential demographic disparities in medication use. The insights from this study establish a policy-relevant baseline for tracking services and could significantly inform health-care policies and guide efforts to optimize ADHD treatment approaches.

## Supplemental Material

sj-docx-1-cpa-10.1177_07067437261471748 - Supplemental material for Prevalence, Trends and Patterns of ADHD Medication Use Among Children, Adolescents and Young Adults in Quebec, Canada: A Repeated Cross-Sectional Population-Based Study From 2000 to 2022: Prévalence, tendances et profils d’utilisation des médicaments pour le TDAH chez les enfants, les adolescents et les jeunes adultes au Québec (Canada) : étude populationnelle transversale répétée, 2000-2022 Supplemental material, sj-docx-1-cpa-10.1177_07067437261471748 for Prevalence, Trends and Patterns of ADHD Medication Use Among Children, Adolescents and Young Adults in Quebec, Canada: A Repeated Cross-Sectional Population-Based Study From 2000 to 2022: Prévalence, tendances et profils d’utilisation des médicaments pour le TDAH chez les enfants, les adolescents et les jeunes adultes au Québec (Canada) : étude populationnelle transversale répétée, 2000-2022 by Martin Gignac, Alvine A.K. Fansi, Carlotta Lunghi, Fatoumata Binta Diallo, Louis Rochette, Victoria Massamba, Helen-Maria Vasiliadis, Elham Rahme, Samuele Cortese and Alain Lesage in The Canadian Journal of Psychiatry
