# Supplementary material for: Prevalence, Trends and Patterns of ADHD Medication Use Among Children, Adolescents and Young Adults in Quebec, Canada: A Repeated Cross-Sectional Population-Based Study From 2000 to 2022: Prévalence, tendances et profils d’utilisation des médicaments pour le TDAH chez les enfants, les adolescents et les jeunes adultes au Québec (Canada) : étude populationnelle transversale répétée, 2000-2022
Source: Can J Psychiatry. 2026 Aug 26:07067437261471748. Online ahead of print. doi: 10.1177/07067437261471748 (PMC13530039; doi:10.1177/07067437261471748)
Supplement: Supplementary file 1 [file sj-docx-1-cpa-10.1177_07067437261471748.docx]

**Supplementary Material**

**Prevalence, Trends and Patterns of ADHD Medication Use Among Children, Adolescents and Young Adults in Quebec, Canada: A Repeated Cross-Sectional population-based study from 2000 to 2022**

Martin Gignac, Alvine Fansi, Carlotta Lunghi, Fatoumata Binta Diallo, Louis Rochette, Victoria Massamba, Helen-Maria Vasiliadis, Elham Rahme, Samuele Cortese, Alain Lesage

**Figure S1** Flow diagram showing the mean annual number and percentage of individuals aged 1–24 years in Québec, stratified by coverage under the public drug plan and by the presence of an ADHD diagnosis and/or an ADHD medication claim. Percentages are calculated within each coverage stratum.

ADHD diagnosis denotes ≥1 ADHD diagnosis recorded during the fiscal year; ADHD medication denotes ≥1 dispensing claim for an ADHD medication during the fiscal year. QICDSS: Quebec Integrated Chronic Disease Surveillance System. PDP: public drug plan.

*****: Total PDP coverage, namely individuals insured continuously for the entire fiscal year (365 days, no interruptions).

**#:** Partial PDP coverage = individuals insured for <365 days during the fiscal year.

**±** indicates the mean annual number and percentage of individuals across the study period.

**Figure S2** Age-adjusted annual prevalence of ADHD medication prescribing among individuals aged 1 to 24 years, fully covered by the public prescription drug insurance plan, according to material (**A**) and social (**B**) deprivation at birth, by fiscal year from 2000-2001 to 2022-2023

**Figure S3** Age-standardized distribution of annual ADHD prescription claims among 1-24 individuals fully covered by the public prescription drug plan. (**A)** Distribution by medication class (stimulants vs. non-stimulants) and (**B**) by medication type (methylphenidate-based, amphetamine-based, atomoxetine-based, and guanfacine-based medications).

Note: This analysis measures the volume of prescriptions rather than the number of users. The denominator is the total number of ADHD prescriptions claimed in the fiscal year; the numerator is the number of claims for the indicated class or type.

**Figure S4** Crude prevalence rates of ADHD medication use among individuals aged 1 to 24 years fully covered by the public drug plan, by age group. **A.** Stimulants and **B.** Non stimulants

**Figure S5** Crude prevalence rates of ADHD medication use among individuals aged 1 to 24 years fully covered by the public drug plan, by age group and sex. **A.** Stimulants and **B.** Non stimulants

**Figure S6** Crude prevalence rates of ADHD medication use among individuals aged 1 to 24 years covered by the public drug plan, by age group and sex according to type (methylphenidate-based, amphetamine-based, atomoxetine-based, and guanfacine-based medications)

**Supplementary Table S1** Age-adjusted prevalence rates of ADHD medication use among individuals aged 1 to 24 years fully covered by the public drug plan, by (**A**) ADHD class (stimulants vs. non-stimulants) and (**B**) medication type (methylphenidate-based, amphetamine-based, atomoxetine-based, and guanfacine-based medications)

| **Year** | **Amphetamine-based** | | **Methylphenidate-based** | | **Atomoxetine** | | **Guanfacine** | |
| --- | --- | --- | --- | --- | --- | --- | --- | --- |
|  | **Prevalence** | **(99% CI)** | **Prevalence** | **(99% CI)** | **Prevalence** | **(99% CI)** | **Prevalence** | **(99% CI)** |
| 2000-2001 | 0.16 | (0.15-0.17) | 1.91 | (1.87-1.95) | 0 |  | 0 |  |
| 2001-2002 | 0.22 | (0.20-0.23) | 2.11 | (2.07-2.16) | 0 |  | 0 |  |
| 2002-2003 | 0.31 | (0.29-0.33) | 2.37 | (2.32-2.42) | 0 |  | 0 |  |
| 2003-2004 | 0.40 | (0.38-0.42) | 2.60 | (2.54-2.65) | 0 |  | 0 |  |
| 2004-2005 | 0.51 | (0.48-0.53) | 2.67 | (2.61-2.72) | 0 |  | 0 |  |
| 2005-2006 | 0.54 | (0.51-0.56) | 2.78 | (2.73-2.84) | 0.05 | (0.04-0.06) | 0 |  |
| 2006-2007 | 0.60 | (0.57-0.62) | 2.91 | (2.85-2.96) | 0.19 | (0.17-0.20) | 0 |  |
| 2007-2008 | 0.68 | (0.65-0.71) | 3.07 | (3.01-3.13) | 0.30 | (0.28-0.32) | 0 |  |
| 2008-2009 | 0.80 | (0.77-0.83) | 3.34 | (3.28-3.40) | 0.39 | (0.37-0.41) | 0 |  |
| 2009-2010 | 0.92 | (0.88-0.95) | 3.63 | (3.56-3.69) | 0.47 | (0.45-0.49) | 0 |  |
| 2010-2011 | 1.03 | (1.00-1.07) | 3.96 | (3.89-4.03) | 0.52 | (0.49-0.54) | 0 |  |
| 2011-2012 | 1.31 | (1.27-1.35) | 4.23 | (4.15-4.30) | 0.54 | (0.52-0.54) | 0 |  |
| 2012-2013 | 1.67 | (1.62-1.71) | 4.41 | (4.33-4.48) | 0.57 | (0.55-0.60) | 0 |  |
| 2013-2014 | 2.00 | (1.95-2.05) | 4.56 | (4.49-4.64) | 0.60 | (0.57-0.62) | 0.03 | (0.03-0.04) |
| 2014-2015 | 2.31 | (2.25-2.36) | 4.79 | (4.72-4.87) | 0.59 | (0.56-0.62) | 0.22 | (0.20-0.24) |
| 2015-2016 | 2.63 | (2.57-2.68) | 5.10 | (5.02-5.18) | 0.60 | (0.57-0.62) | 0.39 | (0.36-0.40) |
| 2016-2017 | 2.95 | (2.89-3.01) | 5.31 | (5.23-5.39) | 0.62 | (0.59-0.65) | 0.49 | (0.46-0.51) |
| 2017-2018 | 3.13 | (3.07-3.19) | 5.42 | (5.34-5.50) | 0.62 | (0.59-0.65) | 0.59 | (0.57-0.62) |
| 2018-2019 | 3.16 | (3.10-3.22) | 5.36 | (5.28-5.44) | 0.59 | (0.57-0.62) | 0.63 | (0.61-0.66) |
| 2019-2020 | 3.14 | (3.08-3.20) | 5.01 | (4.93-5.08) | 0.54 | (0.51-0.56) | 0.67 | (0.64-0.70) |
| 2020-2021 | 3.09 | (3.03-3.15) | 4.80 | (4.73-4.88) | 0.53 | (0.50-0.55) | 0.72 | (0.69-0.75) |
| 2021-2022 | 3.23 | (3.17-3.29) | 4.90 | (4.82-4.97) | 0.50 | (0.47-0.52) | 0.75 | (0.72-0.78) |
| 2022-2023 | 3.18 | (3.12-3.24) | 4.70 | (4.63-4.77) | 0.47 | (0.45-0.49) | 0.75 | (0.72-0.78) |

CI: confidence interval
